# Exploring the role of gut microbiota dysbiosis in gout pathogenesis: a systematic review

**DOI:** 10.3389/fmed.2023.1163778

**Published:** 2023-05-17

**Authors:** Salman Shirvani-Rad, Niloufar Khatibzade-Nasari, Hanieh-Sadat Ejtahed, Bagher Larijani

**Affiliations:** ^1^Microbiota Research Group, Endocrinology and Metabolism Research Center, Endocrinology and Metabolism Clinical Sciences Institute, Tehran University of Medical Sciences, Tehran, Iran; ^2^Faculty of Medicine, Qeshm International Medical Sciences Branch, Islamic Azad University, Qeshm, Iran; ^3^Young Researchers and Elite Club, Qeshm International Medical Sciences Branch, Islamic Azad University, Qeshm, Iran; ^4^Obesity and Eating Habits Research Center, Endocrinology and Metabolism Clinical Sciences Institute, Tehran University of Medical Sciences, Tehran, Iran; ^5^Endocrinology and Metabolism Research Center, Endocrinology and Metabolism Clinical Sciences Institute, Tehran University of Medical Sciences, Tehran, Iran

**Keywords:** gout, gut microbiota, microbiome, dysbiosis, hyperuricemia

## Abstract

**Objectives:**

Gut dysbiosis is believed to be one of the several mechanisms that are involved in the pathogenesis of gout. This systematic review aimed to summarize the role of gut dysbiosis in gout disease and uncover the underlying mechanisms.

**Methods:**

A comprehensive search was conducted on PubMed, Web of Science, and Scopus databases up to October 2021. Animal studies and human observational studies, including case-control, cross-sectional, and cohort studies assessing the association between gut microbiota composition and gout were included. The quality of included studies has been evaluated using the Newcastle–Ottawa Quality Assessment scale (NOS) and the SYRCLE's risk of bias tool.

**Results:**

Initially, we found 274 studies among which 15 studies were included in this systematic review. Of them, 10 studies were conducted on humans and 5 studies were conducted on animals. Increased abundance of *Alistipes* and decreased abundance of *Enterobacteriaceae* alters purine metabolism, thereby aggravating gout condition. Moreover, a higher abundance of *Phascolarctobacterium* and *Bacteroides* in gout modulates enzymatic activity in purine metabolism. Butyrate-producing bacteria such as *Faecalibacterium, prausnitzii, Oscillibacter, Butyricicoccus*, and *Bifidobacterium* have higher abundance in healthy controls compared to gout patients, suggesting the anti-inflammatory and anti-microbial role of short-chain fatty acids (SCFAs). Lipopolysaccharides (LPS)-releasing bacteria, such as *Enterobacteriacea*e, *Prevotella*, and *Bacteroides*, are also involved in the pathogenesis of gout disease by stimulating the innate immune system.

**Conclusion:**

Exploring the role of gut dysbiosis in gout and the underlying mechanisms can help develop microbiota-modulating therapies for gout.

## Introduction

Gout is an inflammatory arthritis disease caused by purine metabolism disorder and characterized by elevated levels of serum uric acid (SU) and deposition of monosodium urate (MSU) in and around the joints (1–3). Due to the changes in diet and lifestyle, the prevalence and incidence of gout have gradually increased worldwide (1, 4, 5). Hyperuricemia (HUA) is a major risk factor for MSU crystal deposition and gout complications such as acute gouty arthritis, joint deformity, and uric acid nephropathy (6, 7). Both gout and HUA are critical risk factors for different metabolic diseases such as hypertension, chronic kidney disease (CKD), obesity, cardiovascular disease, stroke, and diabetes mellitus (5, 8, 9).

Gut microbiota refers to the community of microorganisms residing in human intestines. Gut microbiota plays crucial roles in the physiological functions of the gastrointestinal tract such as food digestion, anti-microbial defense, and metabolism (2, 10–13). Several metabolomics and metagenomics studies described the association between gut dysbiosis and gout to differentiate between gout patients and healthy individuals and provide a novel insight for disease treatment (8, 11).

HUA is associated with purine abnormal metabolism and decreased UA excretion. In a healthy person, about 70% of the UA excretion occurs in the kidney, whereas the remaining is excreted through the intestines and is metabolized by gut microbiota. Several studies have indicated that gut microbiota and their metabolites contribute to purine and UA metabolism (10, 14). However, to date, the mechanisms linking host purine and UA metabolism to gut microbiota are not completely determined. Therefore, recent studies have focused on the involvement of gut microbiota in hyperuricemia to uncover the mediating mechanisms linking gut dysbiosis to gout (1, 2, 7, 15).

Some studies revealed that the abundance of *Bacteroides* is remarkably increased in gout patients while the abundance of *Faecalibacterium* is decreased. These alterations in the composition of gut microbiota are the hallmark of gout disease and can accelerate disease progression (1, 11). Another study revealed that gut dysbiosis can alter intestinal immunity and increase bacterial penetration into the systemic circulation, thereby inducing a systemic inflammatory response and aggravating gout disease (16, 17). Moreover, studies have found that gut microbiota is an important target for treating HUA by enhancing purine and UA catabolism, increasing UA excretion, and modulating intestinal inflammatory response (8, 15, 18). In this systematic review, we aimed to provide a better insight into the association of gut dysbiosis with gout disease.

## Methods

This systematic review was done according to the Preferred Reporting Items for Systematic Reviews and Meta-analysis (PRISMA) statement (19).

### Search strategy

PubMed, Web of Science, and Scopus databases were searched for observational studies on the relationship between gout and changes in gut microbiota composition up to October 2021. The search terms were “gout” OR “hyperuricemia” OR (“arthritis” AND “gouty”) AND “microbiome” OR “microbiota” OR “dysbiosis” OR “gut microbial composition” OR “intestinal microbial composition” OR “Fecal microbial composition” OR “gut bacterial composition” OR “intestinal bacterial composition” OR “Fecal bacterial composition” OR “intestinal microflora” OR “gut microflora.” Furthermore, the reference list of related review articles in this field was screened. All retrieved articles were checked and duplicates were removed manually by two independent researchers.

### Eligibility criteria and study selection

Initially, all documents were screened according to the titles and abstracts by two independent researchers. Thereafter, the full-text version of the articles was reviewed based on the inclusion and exclusion criteria. In case of any disagreements between the two researchers, they continued discussing until reaching a consensus.

Animal studies or human observational studies including case-control, cross-sectional, and cohort studies assessing the correlation between gut microbiota and gout were included.

Regarding “PECOS” of this systematic review:

Population (P): gout patients
Exposure (E): microbiota dysbiosis
Controls (C): healthy subjects
Outcomes (O): gout symptoms and disease intensity
Study design (S): observational

Reviews, case reports, experimental studies, interventional studies, protocols, conference papers, and letters to the editor were excluded.

### Data extraction

Data were extracted from both human and animal studies by two independent researchers. For animal studies, authors' names, publication year, characteristics of animals (sample size, age, and sex), HUA induction method, microbiota and biochemical analysis method, and microbiota and biochemical changes in the HUA group were collected. For human observational studies, authors' names, country and year of publication, participants' characteristics (sample size and age), diagnostic tools for gout, medications, microbiota analysis method, main findings regarding microbiota profile, and biochemical changes were collected.

### Quality assessment

The Newcastle–Ottawa Quality Assessment scale (NOS) has been used to evaluate the quality of cohort and case-control studies, and its modified version has been used to evaluate the quality of cross-sectional studies (20, 21). The maximum score was 9 for cohort and case-control studies and 7 for cross-sectional studies. The quality of studies was defined good if the studies got 3 or 4 stars in the selection domain AND 1 or 2 stars in the comparability domain AND 2 or 3 stars in the outcome/exposure domain. Fair quality was defined as 2 stars in the selection domain AND 1 or 2 stars in the comparability domain AND 2 or 3 stars in the outcome/exposure domain. Poor quality was defined as 0 or 1 star in the selection domain OR 0 stars in the comparability domain OR 0 or 1 stars in the outcome/exposure domain (20). Besides, the quality of animal studies was measured based on the SYRCLE's risk of bias tool (22).

All procedures including searching, study selection, data extracting, and quality assessment were conducted by two separate and independent researchers and they discussed the conflicting points until they reached a consensus.

## Results

**Figure 1:**
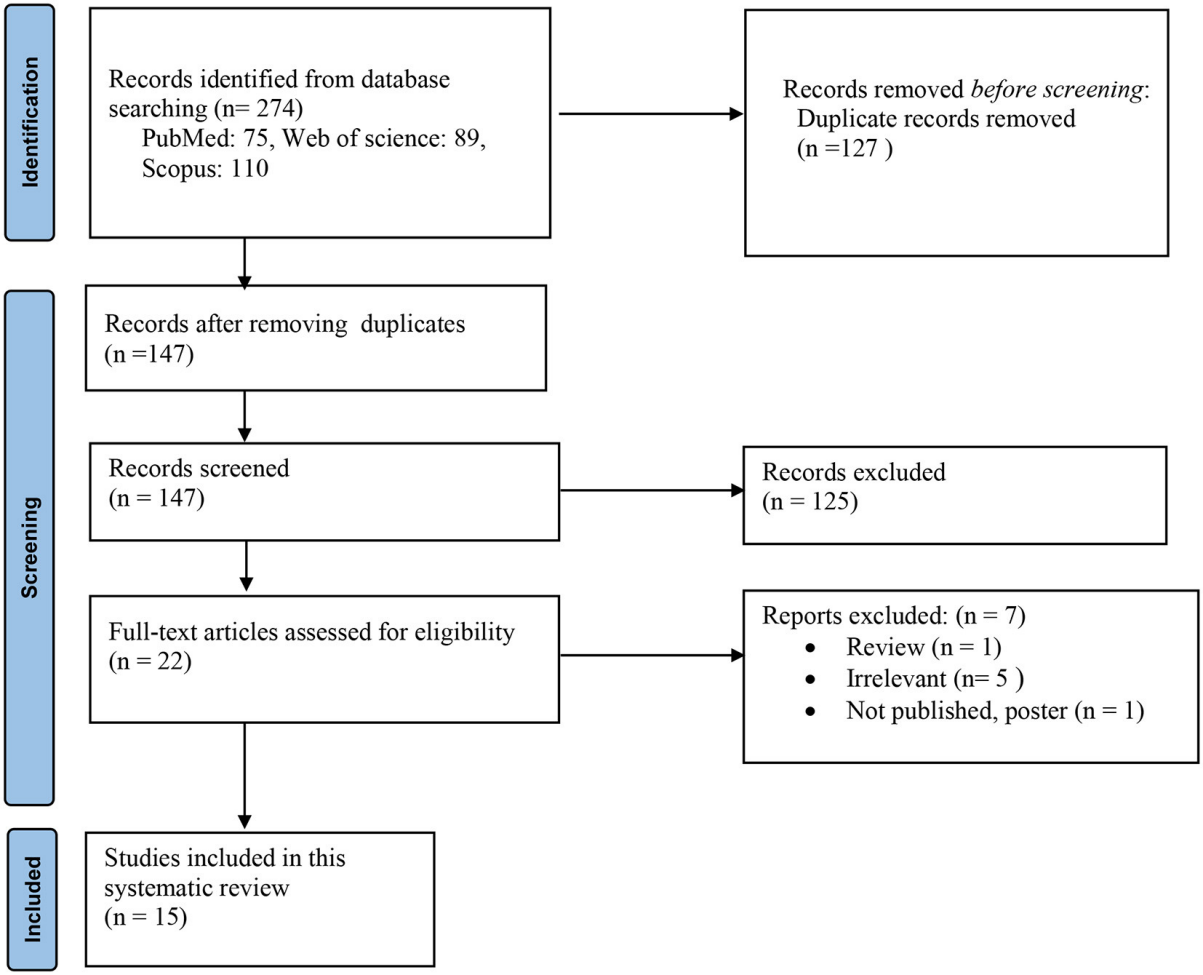
Flow diagram of the study selection process.

Primarily, we identified 274 studies, and 127 articles were removed because of duplication. Then, 147 studies were screened using their title/abstract and full text. Finally, 15 studies were included in this systematic review (Figure 1).

### Human studies

Data from ten included articles are presented in Table 1. Among them, 8 were case-control (2–5, 8, 10, 11, 14), 1 was a cohort (1) and 1 was a cross-sectional study (24). Totally, there were 477 participants with gout disease and 494 participants as the control group. Henson et al. (5) Liu et al. (11) and Yang et al. (24) included both genders whereas Ning et al., Shao et al., and Xing et al. only included men (2, 3, 10). The gender of participants was not mentioned in other studies (1, 4, 8, 14). The average age of participants ranged from 28 to 75 years (1–5, 10, 11, 24). Two studies did not mention the age of participants (8, 14).

**Table 1:** Alteration in microbiota composition and biochemical variables in human studies.

**Study**	**Country**	**Type of study**	**Sample size**		**Age**		**Diagnostic tool**	**Medication**	**Microbiota analysis**	**Biochemical analysis**	**Main study findings (Microbiota profile)**	**Biochemical changes**	**Quality assessment score**
			**Gout patients**	**Control**	**Gout patients**	**control**							
Méndez-Salazar et al. (14)	Mexico	Case-control	58 (33 with a tophi and 25 without tophi)	53	Not mentioned	Not mentioned	2015 ACR/EULAR (23)	No antibiotics, or anti-parasitic, other medication were not mentioned	Sequencing hypervariable V3–V4 regions of the bacterial 16S rRNA genes (Illumina Miseq platform)	Analysis of blood sample	**Richness indices**: Chao1 ↑ Observed species ↑ ACE ↑ in controls. **In controls Genera**: *Ruminococcus_ 1* ↑, *Clostridium_ sensu_ stricto_ 1* ↑*Oscillibacter* ↑, *Butyricicoccus* ↑*, Ruminococcaceae_ UCG_ 010* ↑, *Bifdobacterium* ↑, *Lachnospiraceae_ ND3007_ group*↑, *Haemophilus* ↑ and *Ruminococcaceae_ UGC_ 013* ↑**In gout without tophi Genera**: *Phascolarctobacterium* ↑, *Akkermansia* ↑, *Bacteroides* ↑ and Ruminococcus_ gnavus_ group ↑ from controls and *Lachnospira* ↑, *Erysipelotrichaceae_ UCG_ 003* ↑, *Roseburia* ↑, *Ruminococcaceae_ UGC_ 013* ↑ *Erysipelotrichaceae_ UCG_ 003* ↑ and *Akkermansia* ↑ from gout with tophi **In gout with tophi Phylum**: Proteobacteria ↑ **Genera:** *Escherichia-Shigella* ↑ From controls and **Genera:** *Sarcina* ↑,	**In controls:** Urea carboxylase ↑ and urease accessory protein ↑ **In gout without tophi:** glycine reductase complex component B subunits alpha, beta and gamma ↑ and Glycine dehydrogenase subunit ↑ **In gout with tophi:** Vitamin B12 (permease protein and substrate binding protein) ↑, Nucleoside permease ↑, Xanthine phosphorylases ↑, Nucleoside phosphorylases ↑, Methionine transaminase ↑,	**6/9**
											*Rikenellaceae_ RC9* ↑, *Lachnospiraceae_ NK4B4* ↑ and *Lachnospiraceae_ ND3007* ↑ From gout without tophi.	Glycine cleavage system transcriptional repressor ↑, Xanthine dehydrogenase iron-sulfur-binding subunits↑, 5-hydroxyisourate hydrolase ↑, (S)-ureidoglycine aminohydralase ↑ and purine nucleosidase ↑	
Chu et al. (4)	China	Nested case-control	102 (77 discovery gout + 25 validation gout)	86 (63 discovery control + 23 validation control)	Discovery= 39.9 ± 12.9 Validation= 41.9 ± 14.4	Discovery= 40.0 ± 12.1 Validation= 38.3 ± 13.6	2015 ACR/EULAR (23)	No antibiotics and glucocorticoid use within 3 months and 1 month for patients and controls	Metagenomic shotgun sequencing	Analysis of blood sample	**Phylum**: Bacteroidetes ↑, Fusobacteria ↑ Proteobacteria ↓**Species:** 3 species of *Bacteroides* **↑** 13 species of *Prevotella* ↑ 4 species of *Fusobacterium ↑*	ESR ↑, CRP ↑, SCr ↑ and SUA ↑ In gout patients than controls	**5/9**
Yang et al. (24)	China	Cross-sectional	45 patients with AH 45 cases in the control group (57: Male, 33: Female)		60 (49–66.25 years)		Index of the blood uric acid was more than 360 μmol/L (for woman), or more than 420 μmol/L (for man) in two fasting blood uric acid determinations on separate days	No antibiotic antibiotics or probiotics within 3 months	PCR amplification of the V3-V4 region of 16S rRNA genes	Analysis of blood lipid, routine blood testing parameters, blood biochemical analysis, liver function parameters and renal function parameters	**α-diversity:** Chao1 index ↑ Ace index ↑ Shannon index ↑ In AH group **Genera:** *unclassified_ Ruminococcaceae* ↑*, Alistipes* ↑*, Dialister* ↑*, unidentified_ Ruminococcaceae* ↑*, Roseburia* ↑*, Gemmiger* ↑, and *Faecalibacterium* ↑ in AH group *unclassified_ Enterobacteriaceae* ↑, *Bifidobacterium* ↑, *Klebsiella* ↑, *Ruminococcus* ↑, *unidentified_ Lactobacillales* ↑, *unclassified_ Enterococcaceae* ↑, *Eubacterium* ↑, *unidentified_ Enterobacteriacea*e ↑, and *Clostridium* ↑ in control group	NA	**6/7**
Lin et al. (8)	China	Nested case-control	38 patients 26 healthy participants Both gout patients and healthy controls were from local inhabitants with the same gender and similar age				(23), elevation of uric acid and typical clinical manifestations	No anti-gout drugs, steroids, proton pump inhibitors, nonsteroidal anti-inflammatory drugs, Traditional Chinese medicine or any other drugs in three months before admission to the study.	metagenomic shotgun sequencing and 16S rRNA genes sequencing	N/A	**Phylum:** Actinobacteria ↑ in healthy controls and Firmicutes ↑ in untreated patients from HCs **Genera:** *Fecalibacterium* ↑, *Lachnospiraceae Clostridium* ↑, *Roseburia* ↑, *Cytophaga* ↑, *Ruminococcaceae Clostridium* ↑, *Alistipes* ↑, *Pseudomonas* ↑, *Butyricicoccus* ↑, *Clostridiaceae Clostridium* ↑, *Sporobacter* ↑, *Campylobacter* ↑, *Desulfotomaculum* ↑, *Halomonas and Succinispira* ↑ in untreated patients from HCs *Millisia* ↑, *Bifidobacterium* ↑, *Paracoccus* ↑, *Aeromonas* ↑, *Enterococcus* ↑ and *Leifsonia* ↑ in HCs	NA	**6/9**
Henson et al. (11)	USA	Case-control	41 (24: Male, 17: Female)	42 (25: Male, 17:Female)	49.4	48.7	clinical symptoms and elevated blood uric acid levels	Not mentioned	16S rRNA gene amplicon library sequencing.	N/A	*Faecalibacterium* ↑ in the healthy samples	BUN ↑ In gout patients Butyrate ↑, L-lactate ↑, L-cysteine ↑, L-methionine ↑, H2S ↑, L-isoleucine ↑, 3-methyl-2-oxovaleric acid ↑, L-histidine ↑ and L-tyrosine ↑ In low gout cluster Alanine ↑, H2 ↑, Isobutyrate ↑, Isocaproate ↑ and Isovalerate ↑ In high gout cluster	**4/9**
Ning et al. (3)	China	Nested case-control	30 (Male)	30 (Male)	45.86	41.36	2015 ACR/EULAR (23)	No antibiotics within 1 month, other mediction were not mentioned	PCR Amplification of the Bacterial 16S rRNA V3–V4 Region and Illumina Pyrosequencing	N/A	**α-diversity:** ACE index ↓ Chao1 index ↓ Shannon index ↓ Simpson index ↓ observed-species index ↓ in gout group **Phylum:** Firmicutes ↑, Actinobacteria ↑ and Proteobacteria ↓ in gout patients **Genera:** *Corynebacterium_ 1* ↑, *Prevotella* ↑ and *Novosphingobium* ↓, *Derxia* ↓, *Curvibacter* ↓, *Methylobacterium* ↓, *Caulobacter* ↓, *Skermanella* ↓ *unidentified_ Chloroplast* ↓ and *Rikenellaceae_ RC9_ gut_ group* ↓ in gout patients	UA serum levels ↑ and BUN ↑ In gout patients	**7/9**
Liu et al. (5)	China	Nested case-control	12 gout patients (8: Male, 4: Female) + 11 (9: Male, 2: Female) HUA patients	19 (15: Male, 4: Female)	54.42 years for gout patients 53.5 years for HUA patients	55.95	NA	Only subjects with a non-smoking history	16S rDNA sequencing (Illumina HiSeq 2000 platform)	N/A	**Species:** *Prevotella intermedia* ↑and *Streptococcus anginosus* ↑ in HUA and gout patients *Serratia marcescens* ↑ in HCs	NA	**2/9**
Shao et al. (2)	China	Case-control	26 (Male)	26 (Male)	43.60	39.42	The clinical diagnosis and blood examination reports	Patients: No medical treatment within 1 month of study participation, healthy controls: No antibiotics within 1 month of this study	PCR amplification of the V3-V4 region of 16S rRNA genes	^1^H NMR spectra assaying	**α-diversity:** Chao1 ↓, Observed species ↓, Simpson ↓ and Shannon ↓ in gout patients **In gout patients Phylum:** Chloroflexi ↑, Bacteroidetes ↑**Class:** *Erysipelotrichia* ↑, *Negativicutes* ↑, *Anaerolineae* ↑ and *Bacteroidia* ↑**Order:** *Bacteroidales* ↑, *Anaerolineales* ↑, *Selenomonadales* ↑, *Corynebacteriales* ↑and *Erysipelotrichales* ↑ **Family:** *Nocardiaceae* ↑, *Bacteroidaceae* ↑, *Anaerolineaceae* ↑, *Porphyromonadaceae* ↑, *Erysipelotrichaceae* ↑ and *Vibrionaceae* ↓ **Genus:** *Rhodococcus* ↑, *Erysipelatoclostridium* ↑ and *Photobacterium* ↓, *Vibrio* ↓, *Coprococcus 3* ↓, *Lachnospiraceae NC2004 group* ↓, *Lachnospiraceae UCG_ 005* ↓, *Ruminococcaceae NK4A214 group* ↓ and *Ruminococcaceae UCG_ 011* ↓	ESR ↑, UA ↑ and BUN ↑ Alanine ↑, Glycine ↑, Taurine ↑, Succinate ↑, Acetate ↑, α-glucose ↑, β-glucose ↑, α-xylose ↑ valine ↓, asparagine ↓, aspartate ↓, citrulline ↓, phenylalanine ↓ and α-ketoisocaproate ↓ In gout patients	**5/9**
Guo et al. (1)	China	Cohort	35 gout + 6 validation gout	33 + 9 validation control	32–75 years (validation gout =28-69)	aged 28–70 years (validation control = 28-69)	The analysis of blood uric acid for patients with painful joints	Not mentioned	PCR amplification of the bacterial 16S rRNA genes V1-V3 region and pyrosequencing.	N/A	**Genera:** *Coprococcus* ↑, *Faecalibacterium* ↑, *Alistipes* ↑, *Dialister* ↑, *Robinsoniella* ↑, *Subdoligranulum* ↑, *Odoribacter* ↑ and *Oscillibacter* ↑ in controls *Barnesiella* ↑, *Parasporobacterium* ↑, *Paraprevotella* ↑, *Anaerotruncus* ↑, *Pseudobutyrivibrio* ↑, *Bacteroides* ↑, *Holdemania* ↑ and *Acetanaerobacterium* ↑ in gout patients **Species:** *Faecalibacterium prausnitzii* ↑ and Bifidobacterium ↑ pseudocatenulatum ↑ In healthy individuals *Bacteroides caccae* ↑ and *Bacteroides xylanisolvens* ↑ in gout patients	Blood uric acid value ↑ in gout patients	**5/9**
Xing et al. (10)	China	Case-Control	90 (Male)	94 (Male)	47.5 years (40 to 60 years)	49.19 (40 to 60 years)	ACR (American College of Rheumatology) in 1977	No antibiotics or flora products for 1 month before the sample taking	16S rRNA specific primers of both Bacteroides and Clostridium adopted for the PCR amplification.	Analysis of blood sample	**The Diversity Analysis: Bacteroides** the two groups Shannon–Weaver (H' index) and had no statistical significance **Clostridium** the numbers of Clostridium strips and the H' index were much lower in the gout group than the normal control group with statistical significance **The Clustering Analysis: Bacteroides** the normal samples in clustering analysis gathered into 1–2 clusters and the similarity was high. However, the clusters in the primary gout group distributed more dispersed, less clustering with lower similarity **Clostridium** The clustering analysis result that there were about 2/4–3/4 of the normal control samples gathered into 1–2 clusters, the clusters in the primary group distributed more dispersed, less clustering with lower similarity	**Bacteroides** UA ↑ In gout patients	**7/9**
										Analysis of blood sample		**Clostridium** UA ↑ In gout patients	

NA, not available; HUA, hyperuricemia; HC, healthy controls; ESR, erythrocyte sedimentation rate; UA, uric acid; SCr, serum creatinine; CRP, C-reactive protein; BUN, blood urea nitrogen.

The 2015 version of ACR/EULAR classification criteria, hematologic examination, and clinical symptoms were used to recognize and verify gout disease. Five studies used 2015 ACR/EULAR classification criteria (3, 4, 8, 10, 14), and other studies used hematologic examination (1, 2, 8, 11, 24). In addition to hematologic examination, clinical symptoms were used for diagnosis in 4 studies (1, 2, 8, 11). Diagnostic tool was not defined in one study (5). In 7 studies, participants did not use any medications including antibiotics (2–4, 8, 10, 14, 24), while 3 studies did not mention whether their participants used anti-gout medications or antibiotics (1, 5, 11). All studies analyzed the diversity and composition of microbiota by 16s rRNA gene sequencing (1–3, 5, 8, 10, 11, 14, 24), except one study that used Metagenomic shotgun sequencing (4). Lin et al. also used both 16s rRNA gene sequencing and Metagenomic shotgun sequencing for analyzing microbiota composition and function (8). Four studies targeted the V3–V4 region of 16S rRNA genes (2, 3, 14, 24), whereas Guo et al. targeted the V1–V3 region of 16S rRNA genes (1).

### Gut microbial composition in gout disease

Alterations of gut microbial profile in patients with gout disease are summarized in Table 1. In the study conducted by MéndezSalazar et al. richness indices were increased in the control group. At the genus level, *Ruminococcus_1, Clostridium_sensu_stricto_1, Oscillibacter, Butyricicoccus, Ruminococcaceae_UCG_010, Bifidobacterium, Lachnospiraceae_ND3007_group, Haemophilus* and *Ruminococcaceae_UGC_013* were more abundant in the control group (14). Furthermore, Chu et al. exhibited higher abundance of phylum Bacteroidetes and Fusobacteria and lower abundance of phylum Proteobacteria (4) and Yang et al. revealed increased alpha diversity in the asymptomatic hyperuricemic group compared with controls. At genus level, *unclassified_Ruminococcaceae, Alistipes, Dialister, unidentified_Ruminococcaceae, Roseburia, Gemmiger* and *Faecalibacterium* had higher abundance in the asymptomatic hyperuricemic group, while *unclassified_Enterobacteriaceae, Bifidobacterium, Klebsiella, Ruminococcus, unidentified_Lactobacillales, unclassified_Enterococcaceae, Eubacterium, unidentified_Enterobacteriaceae* and *Clostridium* had lower abundance in the control group (24). Additionally, Lin et al. found that phylum Actinobacteria has higher abundance in healthy controls and Firmicutes has higher abundance in untreated patients than in healthy controls. They also reported that gerenra *Fecalibacterium, Lachnospiraceae Clostridium, Roseburia, Cytophaga, Ruminococcaceae Clostridium, Alistipes, Pseudomonas, Butyricicoccus, Clostridiaceae Clostridium, Sporobacter, Campylobacter, Desulfotomaculum, Halomonas* and *Succinispira* have higher abundance in untreated patients than in healthy controls and *Millisia, Bifidobacterium, Paracoccus, Aeromonas, Enterococcus* and *Leifsonia* have higher abundance in healthy controls than in untreated patients. Moreover, Ning et al. reported that ACE, Shannon, Chao1, Simpson, and observed-species indices decrease in gout patients. This study also revealed an increased abundance of Firmicutes and Actinobacteria and a decreased abundance of Proteobacteria at phylum level in gout patients. At genus level, the study showed higher numbers of *Corynebacterium_1* and *Prevotella* and lower numbers of *Novosphingobium, Derxia, Curvibacter, Methylobacterium, Caulobacter, Skermanella, unidentified, Chloroplast* and *Rikenellaceae_RC9_gut_group* in gout patients (3). Furthermore, Shao et al. showed that Chao1, observed species, Simpson, and Shannon indices decrease in gout patients. In addition, this study exhibited the increased population of phylum Chloroflexi and Bacteroidetes, Class Erysipelotrichia, Negativicutes, Anaerolineae and Bacteroidia, Order Bacteroidales, Anaerolineales, Selenomonadales, Corynebacteriales and Erysipelotrichales and Family Nocardiaceae, Bacteroidacea, Anaerolineaceae, Porphyromonadaceae, Erysipelotrichaceae, Vibrionaceae in gout patients. At genus level, the study reported higher abundance of *Rhodococcus* and *Erysipelatoclostridium* and lower abundance of *Photobacterium, Vibrio, Coprococcus 3, Lachnospiraceae NC2004 group, Lachnospiraceae UCG_005, Ruminococcaceae NK4A214 group* and *Ruminococcaceae UCG_011* in gout patients (2). Guo et al. found that *Faecalibacterium prausnitzii, Bifidobacterium* and *pseudocatenulatum* were more abundant in healthy individuals whereas *Bacteroides caccae* and *Bacteroides xylanisolvens* were more abundant in gout patients. At genus level, this study also reported higher abundance of *Coprococcus, Faecalibacterium, Alistipes, Dialister, Robinsoniella, Subdoligranulum, Odoribacter*, and *Oscillibacter* in healthy controls and higher abundance of *Barnesiella, Parasporobacterium, Paraprevotella, Anaerotruncus, Pseudobutyrivibrio, Bacteroides, Holdemania*, and *Acetanaerobacterium* in gout patients (1). Xing et al. performed diversity analysis and clustering analysis on genera *Bacteroides* and *Clostridium*. Compared with normal cases, the number of bands and Shannon–Weaver (H') of *Clostridium* but not *Bacteroides* significantly decreased in patients with primary gout. Furthermore, the intra-group and inter-group similarity of both *Bacteroides* and *Clostridium* were lower (10).

Except two studies, all other studies conducted biochemical analysis (8, 24). In the study conducted by Méndez-Salazar et al., the level of urea carboxylase and urease accessory protein were positively correlated in the control group. Compared with the control group, gout patients without tophi had higher levels of glycine reductase complex component B subunits alpha, beta, and gamma and glycine dehydrogenase subunit, while gout patients with tophi had higher levels of vitamin B12, nucleoside permease, xanthine phosphorylases, nucleoside phosphorylases, methionine transaminase, glycine cleavage system, transcriptional repressor, xanthine dehydrogenase, iron-sulfur-binding subunits, 5-hydroxyisourate hydrolase, (S)-ureidoglycine aminohydralase, and purine nucleosidase (14). Three studies reported higher levels of uric acid in gout patients (2, 4, 10). Besides, Chu et al. and Shao et al. studies observed higher levels of erythrocyte sedimentation rate and blood urea nitrogen in gout patients (1, 2, 4).

Quality assessment of the included human studies by the Newcastle–Ottawa Quality Assessment scale (20, 21) revealed that only 1 study had good quality (total score: 7) (10), 5 studies had fair quality (total core: 5–7) (1, 3, 8, 14, 24) and 4 studies had poor quality (2, 4, 5, 11) (Table 1).

### Animal studies

Data from five animal studies are presented in Table 2. The age of animals ranged from 4 to 26 weeks (7, 15–17, 25). Target species were Wistar rats in one study (7), wild-type mice in two studies (16, 17), and Sprague Dawley rats in two studies (15, 25). Three studies used male animals (7, 15, 25), while the others did not mention the gender of the species (16, 17). HUA induction methods were different among studies. One study used 100 mg/Kg/day of yeast-rich forage and purine (7). Other studies used 10% yeast and 15% adenine diet (25) and a high-fat diet containing 10% yeast extract (15) while some studies did not mention the details (16, 17). These studies investigated the association between gut microbiota composition and gout disease (7, 15–17, 25). In all studies, microbiota diversity was analyzed by 16srRNA gene sequencing, using frozen fecal samples (7, 15–17, 25). Three studies targeted the V3–V4 region of 16S rRNA genes (7, 15, 25), while 2 studies targeted the V1–V3 region of 16S rRNA genes (16, 17).

**Table 2:** Alterations in microbiota composition and biochemical variables in HUA animal models.

**Study**	**Sample size**		**Age**	**HUA inducing method**	**Analysis**		**Microbiota changes in HUA group**	**Biochemical changes in HUA group**
	**Hyperuricemia**	**Control**			**Microbiota diversity analysis**	**Biochemical analysis**		
Liu et al. (7)	Twenty nine male wister rats	19 male wister rats	6 weeks old	yeast-rich forage/purine at 100 mg/ (Kg _ d)	16S rRNA gene Sequencing and amplification V3-V4 variable regions/16S rDNA gene Sequencing (frozen fecal sample)	Analysis of plasma biochemical indicators	**α-diversity:** Shannon index ↑**Phylum:** Firmicutes ↑ and Actinobacteria ↓**Genera:** *Prevotella ↓, Anaerovibrio ↓, Alloprevotella ↓, Barnesiella ↓, Clostridium_XlVa* ↑, *Flavonifractor* ↑, *Phascolarctobacterium ↑, Clostridium_XVIII* ↑, *Parabacteroides* ↑, *Robinsoniella* ↑, *Subdoligranulum* ↑, *Catabacter* ↑, *Blautia* ↑, *Bacteroides* ↑, *Olsenella* ↑, *Vallitalea* ↑, *Christensenella* ↑ and *Insolitispirillum* ↑	**Plasma analysis:** UA *↑*, BUN *↑*, Cr *↑*, and TC ↑
LV et al. (16)	Six homozygous mice (UOX-/-)	Six wild type mice (WT)	15 weeks old	Preclinical induced HUA	16S rRNA gene SequencingV1-V3 region (frozen fecal sample)	Analysis of plasma biochemical indicators/Hematoxylin-eosin and immunohistochemical analysis (tissue sample)/ELISA (serum and parenteral tissues)	**Genera**: *Bacteroides ↑,Alloprevotella ↑, Alistipes ↑, Parabacteroides ↑, Clostridium ↓, Lactobacillus ↓, Candidatus ↓* and *Coriobacteriaceae ↓*	**Plasma analysis:** UA ↑, TC ↑, HDL ↑, LDL ↑, **ELISA**: TNF-α ↑ and IL-1β ↑
Pan et al. (25)	Six male SD rats	Six male SD rats	26 weeks old	10% yeast and 0.15% adenine diet	16S rRNA gene Sequencing V3–V4 region (Fecal sample)	Analysis of plasma and urine metabolites	**Diversity**: Shannon index: No significant differences **Phylum**: Actinobacteria ↑, Proteobacteria ↑, Clostridiaceae ↑ and Bacteroidetes ↓**Family level**: *Clostridiaceae* ↑**Genus**: *Flavobacterium* ↑, *Myroides* ↑, *Corynebacterium* ↑, *Alcaligenaceae ↑, Oligella ↑, Blautia ↓* and *Roseburia* ↓	**Plasma analysis:** UA ↑, BUN ↑, and Cr ↑ Plasma aminoacid: Serine glutamate ↑ and Glutamine ↑**Urine analysis:** Phenol *↓*, p-cresol ↓, p-hydroxyphenylacetic acid ↓ and indol-5-ol ↓
Xu et al. (17)	6 hyperuricemia model mice (Hy)	Six wild-type mice (WT)	4 weeks old	Not mentioned	16S rRNA gene Sequencing V1-V3 region (frozen fecal sample)	Biochemical analysis of blood / Hematoxylin-eosin and immunohistochemical analyses (tissue sample) / ELISA (serum and parenteral tissues)	**Diversity**: Shannon index: No significant differences **Phylum**: Firmicutes ↓**Family level:** *Prevotellaceae ↑, Rikenellaceae ↑, Bacteroidaceae ↑* and *Bacteroidales* ↑**Genus:** *Lactobacillus ↑, Clostridium ↑Bacteroides* ↑*and Ruminococcaceae* ↑	**Plasma analysis:** UA ↑, TC ↑, HDL ↑,LDL ↑ and Endotoxins ↑**ELISA**: TNF-α ↑
Yu et al. (15)	Six SD hyperuricaemia rats (Model group), 6 SD allopurinol treated rats (Allopurinol) and 6 SD benzbromarone treated rats (Benzbromarone)	Six male (SD) rats	6-month-old	High-fat feed containing 10% yeast extract/	16S rRNA gene Sequencing and amplification V3-V4 variable Regions (Fecal sample)	Analysis of plasma biochemical indicators	**Diversity**: Shannon index: No significant differences **Phylum**: Bacteroidetes ↑, Lentisphaerae ↑, Firmicutes ↓ and Tenericutes ↓**Genera** *Bacteroides ↑, Parabacteroides ↑, Gemella ↑, Lactococcus ↑, Anaerostipes ↑, Dorea ↑, Anaerotruncus ↑, Allobaculum ↑, Holdemania ↑, Desulfovibrio ↑, Morganella ↑,Proteus ↑, Rothia ↓, Collinsella ↓, Prevotella ↓, Lactobacillus ↓, Streptococcus ↓, Clostridium ↓, Dehalobacterium ↓, Ruminococcus ↓*, and *Anaeroplasma ↓*	**Plasma analysis:** UA *↑*, Cr *↑*, AST ↑ and CHO1↑

HUA, hyperuricemia; SD, sprague Dawley; UA, Uric acid; Cr, creatinine; AST, glutamic oxaloacetic transaminase; CHO1, cholesterol; TC, total cholesterol; HDL, high-density lipoprotein; LDL, low-density lipoprotein; ELISA, enzyme-linked immunosorbent assay; BUN, blood urea nitrogen.

### Gut microbial composition in HUA animal models

At the genus level, studies exhibited an increase in the abundance of *Parabacteroides* and *Bacteroides* (7, 15, 16) and a decrease in the abundance of *Prevotella* (7, 15) and *Ruminococcaceae* in hyperuricemic animals (15, 25). *Four* studies showed conflicting results regarding *Alloprevotella, Clostridium, Lactobacillus* and *Blautia* (7, 15–17, 25). Liu et al. observed a lower abundance of *Alloprevotella*, while Lv et al. reported a higher abundance of this genus (7, 16). Additionally, Liu et al. and Xu et al. revealed a higher frequency of *Clostridium*, whereas Lv et al. and Yu et al. exhibited a lower abundance of this genus (7, 15–17). Lv et al. and Xu et al. also showed an increased number of *Lactobacillus in* hyperuricemic animals, while in Yu et al. reported a decreased abundance of *Lactobacillus* in hyperuricemic animals (15–17). Moreover, Pan et al. found a decreased abundance of *Blautia* and *Ruminococcaceae* when Liu et al. observed an increased abundance of *Blautia*. Xu et al. reported a higher abundance of *Ruminococcaceae* (7, 17, 25). At the phylum level, studies also reported controversial results regarding the abundance of Bacteroidetes and Firmicutes (15, 17, 25). Pan et al. reported a higher abundance of Bacteroidetes in hyperuricemic animals, while in Yu et al. reported a lower abundance of this phylum in hyperuricemic animals (15, 25). Furthermore, Xu et al. showed a lower number of Firmicutes, while Yu et al. reported a higher number of this phylum (15, 17).

Biochemical analysis was also performed in these studies (7, 15–17, 25). All studies used plasma samples for analysis of biochemical indicators except one study that assessed biochemical indicators in both plasma and urine (25). In 2 studies hematoxylin-eosin staining, immunohistochemical analysis, and enzyme-linked immunosorbent assay were performed (16, 17). All studies reported increased levels of uric acid in hyperuricemic animal models (7, 15–17, 25). Three studies reported higher levels of creatinine (7, 15, 25) and 4 studies reported increased levels of total cholesterol in animal models (7, 15–17). In 2 studies, blood urea nitrogen was increased in hyperuricemic mice (7, 16). Pan et al. also reported higher levels of plasma amino acids such as serine glutamate and glutamine. Urine analysis showed lower levels of phenol, p-cresol, p-hydroxypheny lacetic acid, and indol-5-ol in this study (25). An increased level of TNF-α was reported in 2 studies (16, 17). IL-1β did not significantly change in the study conducted by Xu et al. while it significantly increased in the study conducted by Lv et al. (16, 17).

SYRCLE's tool for assessing the risk of bias was used in animal studies (22) and showed that most of the studies had attrition bias, reporting bias, and weakness in sequencing generation in selection bias (Supplementary Table).

## Discussion

### Main findings

This systematic review summarizes gut dysbiosis in gout to illustrate possible correlations. From all studies, just 5 of them reported changes for alpha diversity and richness indices (2, 3, 7, 14, 24). Three studies showed lower richness among gout patients which can be related to intestinal dysbiosis and inflammation in gout (2, 3, 14). In contrast, 2 studies reported higher richness. These contradictions may be related to differences between humans and animals (7) or disease condition (asymptomatic hyperuricemia) (24). At the phylum level, Bacteroidetes abundance increased in three studies (2, 4, 15) and decreased in one study (25). Firmicutes abundance increased in three studies (3, 7, 8) and decreased in 2 studies (15, 17). Actinobacteria abundance decreased in one study (7) and increased in 3 studies (3, 8, 25). In addition, Actinobacteria abundance increased among healthy controls in one study (8). Proteobacteria abundance increased in two studies (14, 25) and decreased in 2 studies (3, 4). At the phylum level, there were contradictions and heterogeneities among the studies. Differences in characteristics of the population, severity of gout, and methodologies could be the reason for these contradictions. At the genus level, increased *Oscillibacter* (1, 14), *Butyricicoccus* (8, 14), and *Dialister* (1, 24) were observed in two studies. Increased abundance of *Bacteroides* among patients was reported in 5 studies (1, 7, 14–16) while *Roseburia* (8, 14, 24), and *Alistipes abundance* (8, 16, 24) were increased in 3 studies. Studies were contradictory regarding *Faecalibacterium* (11). *Faecalibacterium* was higher in AH (1, 24) contrary to its higher frequency identified in controls. In individuals with a higher abundance of *Faecalibacterium*, increased production of butyrate can partly prevent gout. In this study, healthy individuals had 230% higher abundance of *Faecalibacterium* and 550% higher production of butyrate compared with gout patients (11). Additionally, two studies revealed that at the genus level, *Bifidobacterium* was decreased in gout patients (8, 24).

### Underlying mechanisms

Gout is associated with higher levels of uric acid in the blood, which is called hyperuricemia. Many mechanisms are involved in the pathogenesis of hyperuricemia one of which is gut dysbiosis. Accumulating evidence shows that purine metabolism plays a key role in gout by degrading purine to urea or uric acid (1). In gout patients, purine mostly degrades to uric acid causing hyperuricemia (10). Xanthine dehydrogenase degrades purine to uric acid which is highly expressed in gout patients leading to hyperuricemia. Increased abundance of *Alistipes* in AH and its possible effect on purine metabolism, hypothetically may upregulate xanthine dehydrogenase. Therefore, *Alistipes* can be involved in the pathogenesis of gout (1, 24). It is worth noting that *Enterobacteriaceae* acts like allantoinase, an enzyme in purine metabolism, which degrades uric acid to urea (1, 26). Allantoinase is upregulated in treated patients and downregulated in gout patients. Decreased abundance of *Enterobacteriaceae* in gout patients suggests a connection between *Enterobacteriaceae* and Allantoinase. Perhaps, changes in microbial composition can change enzyme levels, thereby ameliorating or aggravating gout. Moreover, *Phascolarctobacterium* and *Bacteroides* had higher abundance in gout patients and convert urate into allantoin. It is proposed that *Phascolarctobacterium* and *Bacteroides* are involved in gout through enzyme modulation (14, 27).

**Figure 2:**
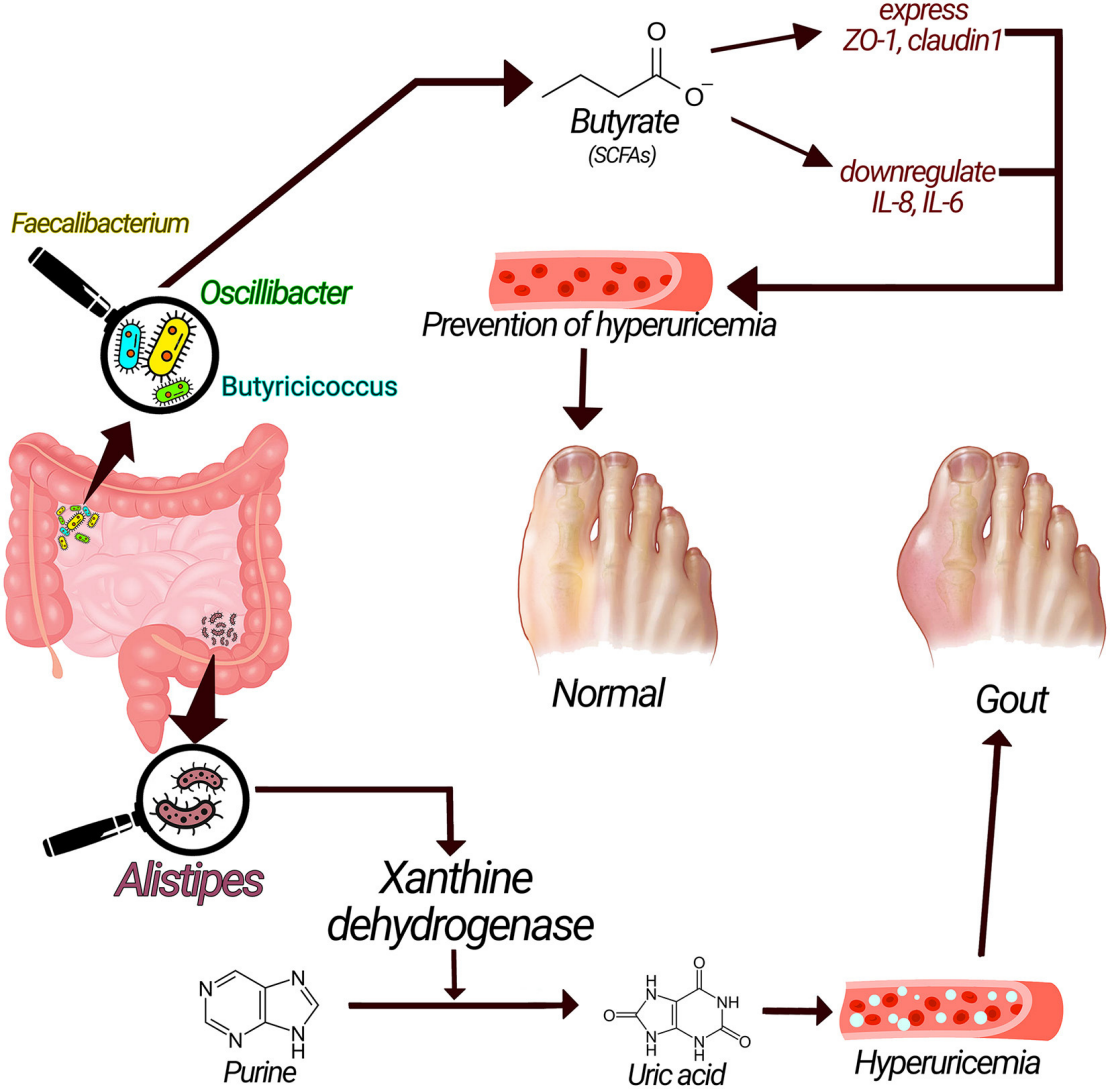
Underlying mechanisms related gut microbiota with gout pathogenesis. Xanthine dehydrogenase produced by *alistipes* could convert purine to uric acid and cause hyperuricemia and increase the risk of gout; on the other hand, SCFAs have anti-inflammatory effect via enhancing the expression of tight junction proteins and have preventive effects on gout.

Acetate, propionate, and butyrate, are short-chain fatty acids (SCFAs) with substantial advantages for health (28). Butyrate possesses protective roles against gout through numerous mechanisms. Butyrate-producing bacteria such as *Faecalibacterium prausnitzii, Oscillibacter*, and *Butyricicoccus* are increased in healthy controls compared to gout patients, suggesting the possible role of SCFAs in gout (1, 14). SCFAs, especially butyrate, maintain the stability and integrity of the epithelial barrier by regulating the expression of tight junction proteins (TJP) such as claudin-1 and Zonula Occludens-1 (ZO-1) (29–31). SCFAs, particularly butyrate, have anti-inflammatory functions by downregulating inflammatory cytokines such as IL-1β, IL-6, and IL-8 or exerting a direct anti-inflammatory effect (Figure 2) (31–33). Moreover, butyrate is an energy source for cells, stimulates the proliferation of healthy cells, and promotes intestinal villus repair (1, 31, 34).

At the genus level, *Bifidobacterium* showed a decreased abundance in gout patients (1, 8, 24, 35). We already know that *Bifidobacterium* has Several protective functions such as reinforcing immune response, acting as a biological barrier (24, 36, 37), preventing aging, and promoting gastrointestinal functionality (8, 38). Furthermore, *Bifidobacterium* can produce butyric acid which has many effects. Furthermore, *Bifidobacterium* can ameliorate constipation and prevent the growth of pathogens (24). The simultaneous presence of *Bifidobacterium* and butyrate-producing bacteria can attenuate inflammation and improve the function of the intestinal barrier (14).

Higher abundance of *Bacteroides* genus (1, 7, 14–16), *Bacteroides caccae* and *Bacteroides xylanisolvens* species (1), *Prevotella* genus and *Prevotella intermedia* species (3, 5) in gout patients (24) supports their role in gout via biosynthesis of LPS or lipid A. LPS is a stimulator of the innate immune system. LPS produced by some species can be transferred into the cytoplasm by interferon-inducible GTPases. The hexa-acylated lipid A component of LPS attaches to caspase 4, caspase 5, caspase 11, and non-canonical NLRP3 inflammasome and activates them (39). The structure of LPS is important in activating the immune system, meaning that only some LPSs can induce the inflammatory response (40). Immune activation depends on the type of acyl chains in LPSs (4). *Prevotella* and *Bacteroides* generally generate LPSs with 4 or 5 acyl chains. Furthermore, they carry two phosphate groups (4, 41, 42) while LPS generated by *Enterobacteriaceae* has six acyl chains and one phosphate group (4, 41). LPSs produced by *Bacteroides* cannot induce the production of cytokines. In contrast, LPS produced by *E. coli* can strongly provoke the production of various cytokines such as IL-10, TNF-α, IL-1β, and IL-6 (43). At the phylum level, Proteobacteria showed contradictory results. It had decreased abundance in two studies (3, 4) and increased abundance in one study (14). It was the most abundant phylum in gout patients and also in healthy controls (3). Some species of Proteobacteria, especially *E.coli*, can generate LPS which activates immune response (4). Proteobacteria downregulates urate oxidase but increases nitrogen fixation capacity in gout (14), which could be an effect of some species other than *E.coli*.

### Therapeutic strategies

Our study aims to reveal microbiota dysbiosis in gout patients and after that, interventional approaches such as probiotics, special dietary patterns, and fecal microbiota transplantation (FMT) may be helpful to practically modulate the gut microbiota composition and amendment of symptoms. So these interventions are needed to be studied in future. Recommended dietary changes decrease SU and lower lipid levels in gout patients (44), and it has been determined that diet partly ameliorates gout by modulating gut microbiota composition (3).

It was observed that *clostridium* can lower uric acid levels, suggesting that *clostridium* can be added to gout-specific probiotic combinations (24, 45). Moreover, SCFAs such as butyrate and acetate have many beneficial effects for gout patients including anti-inflammatory effects and enhancing the intestinal barrier. So, SCFAs-producing bacteria such as *Faecalibacterium prausnitzii, Oscillibacter*, and *Butyricicoccus* could be great targets for intervention (1, 14). Furthermore, using *Bifidobacterium* can improve the anti-inflammatory effects (14).

### Strengths and limitations

The present study has some strengths and limitations. The strength of our systematic review is the comprehensive search of all documents with high accuracy and precision to investigate the correlation between gut microbiota composition and gout. Besides, we evaluated the quality of animal and human studies. Diverse methods used for microbiota analysis, diagnostic tools and outcome measures in different studies are the main limitations of this systematic review which make it challenging to compare and combine the results. Besides, all included studies in this paper were observational in nature so it is hard to establish assured causality but instead correlations were acknowledged. Further studies are needed to generate basic knowledge for clarifying possible underlying mechanisms and any probable causal relationship.

## Conclusion

Microbiota is deeply associated with inflammatory disorders like gout via many mechanisms. Some contradictory results prevent us to determine the role of some special bacterial taxa. There was no clear evidence regarding which bacteria are more protective. Different designs and methodologies of studies could be a reason for those controversies. Generally, studies were different in the eligibility criteria, sample size, diagnostic tools, microbiota and biochemical analysis techniques, which may warrant the controversies. Further studies are needed to improve gout through microbiota-modulating-based therapies.

## Data availability statement

The original contributions presented in the study are included in the article/Supplementary material, further inquiries can be directed to the corresponding author.

## Author contributions

SS-R is contributed as first author. H-SE and BL conceived and coordinated the study. H-SE and BL participated in design of study and cooperated to write final draft. SS-R and NK-N extracted information from the articles and drafted the manuscript. H-SE and SS-R edited the final draft of manuscript. H-SE reviewed the manuscript with a fault-finding view and SS-R did the quality assessment. The title is chosen by BL. All authors read and approved the final manuscript.
