# Supplementary material for: Exploring the role of gut microbiota dysbiosis in gout pathogenesis: a systematic review
Source: Front Med (Lausanne). 2023 May 17;10:1163778. doi: 10.3389/fmed.2023.1163778 (PMC10230090; doi:10.3389/fmed.2023.1163778)
Supplement: Supplementary file 1 [file Table_1.DOCX]

**Additional file 1:**

**Supplementary table. SYRCLE’s tool for assessing risk of bias in animal studies**

| **Study** | **Selection bias** | | |  | **Performance bias** | |  | **Detection bias** | |  | **Attrition bias** |  | **Reporting bias** |
| --- | --- | --- | --- | --- | --- | --- | --- | --- | --- | --- | --- | --- | --- |
|  | **Sequence generation** | **Baseline characteristics** | **Allocation concealment** |  | **Random housing** | **Blinding** |  | **Random outcome assessment** | **Blinding** |  |  |  |  |
| Pan et al., 2019 | Unclear | Low-risk | Low-risk |  | Unclear | High-risk |  | High-risk | High-risk |  | Low-risk |  | Low-risk |
| LV et al.,  2020 | Unclear | High-risk | High-risk |  | High-risk | Unclear |  | Unclear | Unclear |  | Low-risk |  | Low-risk |
| Liu et al.,  2020 | Unclear | Low-risk | High-risk |  | Unclear | High-risk |  | High-risk | High-risk |  | Low-risk |  | Low-risk |
| Xu et al.,  2019 | Unclear | Low-risk | High-risk |  | High-risk | High-risk |  | Unclear | Low-risk |  | Low-risk |  | Low-risk |
| Yu et al.,  2018 | Unclear | Low-risk | High-risk |  | Unclear | High-risk |  | High-risk | High-risk |  | Low-risk |  | Low-risk |
|  |  |  |  |  |  |  |  |  |  |  |  |  |  |
|  |  |  |  |  |  |  |  |  |  |  |  |  |  |
|  |  |  |  |  |  |  |  |  |  |  |  |  |  |
